# Supplementary material for: Machine learning techniques for continuous genetic assignment of geographic origin of forest trees
Source: PLoS One. 2025 Jun 6;20(6):e0324994. doi: 10.1371/journal.pone.0324994 (PMC12143523; doi:10.1371/journal.pone.0324994)
Supplement: S4 Fig — (DOCX) [file pone.0324994.s004.docx]

Supplementary 4 Fig


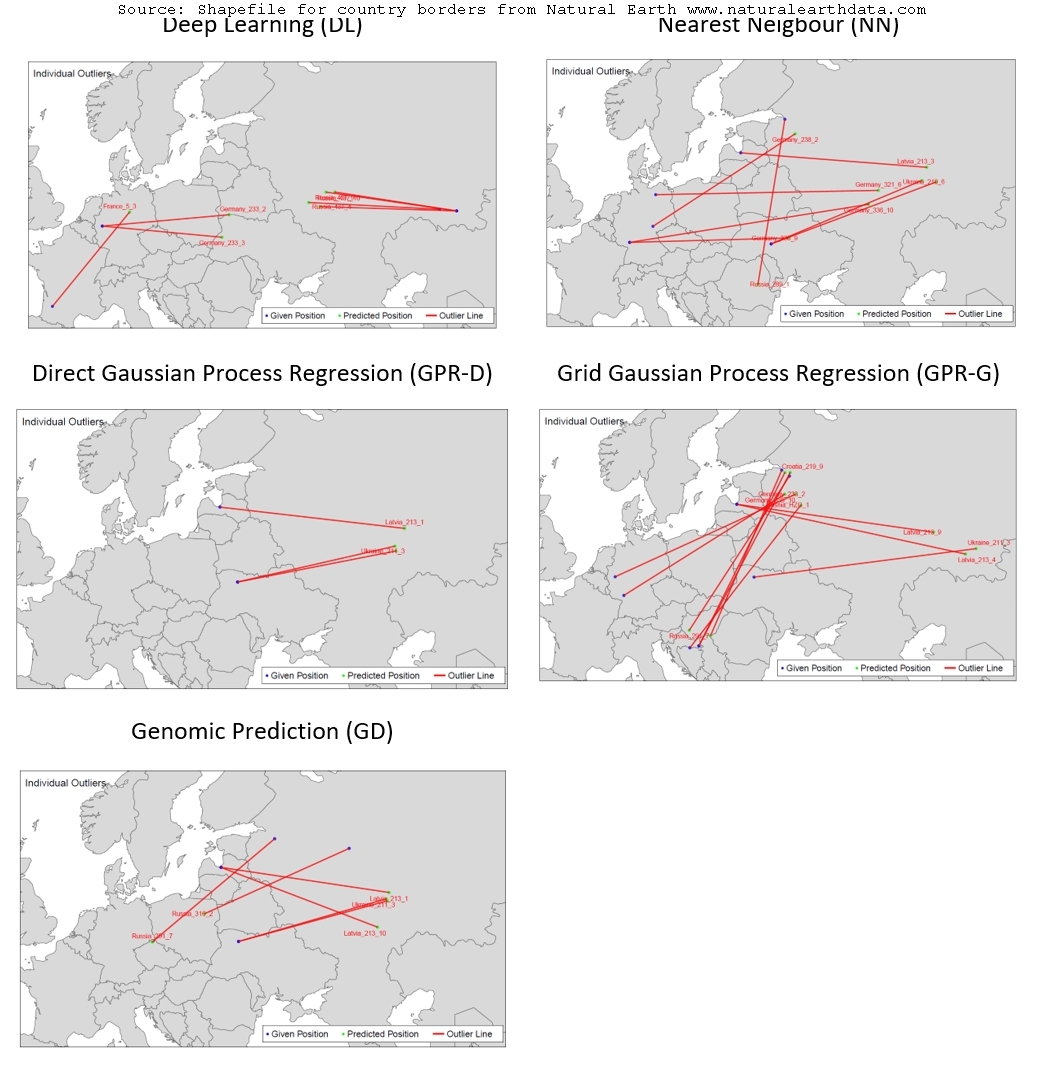


**Sup 4 Fig** Maps showing the individual outliers identified in the oak data set, shapefile of country borders from www.naturalearthdata.com
