# Supplementary material for: Machine learning techniques for continuous genetic assignment of geographic origin of forest trees
Source: PLoS One. 2025 Jun 6;20(6):e0324994. doi: 10.1371/journal.pone.0324994 (PMC12143523; doi:10.1371/journal.pone.0324994)
Supplement: S5 “Supplementary 5.docx” — (DOCX) [file pone.0324994.s005.docx]

Supplementary 5: Comparison of prediction accuracy with previously reported methods

We compared the accuracy of the predicted geographic locations of our five methods: direct gaussian process regression (GPR-D), grid-based gaussian process regression (GPR-G), genomic predictions (GP), nearest neighbour approach (NN) and deep learning (DL) with results obtained by the programs for continuous assignment SPASIBA (Guillot et al. 2016) and SPA (Yang et al. 2012). SPASIBA stands for Spatial Bayesian Interference and is a further development of the SCAT program (Wasser et al. 2004). It uses the allele counts of training samples to model the two-dimensional distribution of the allele frequencies. Other than the SCAT software it does not require Monte Carlo simulations but applies Nested Laplace Approximation for the optimisation of the underlying functions. The spatial ancestry analysis (SPA) also models allele frequencies as continuous functions in geographic space but using logistic functions which were optimized by Newton’s based methods. Unfortunately, both programs SPASIBA and SPA were not updated and we could not apply them to our oak and beech data. Nevertheless, the supplementary material of Guillot et al. (2016) contains results on comparative application of SPASIBA and SPA for a data set of *Arabidopsis thaliana* from Horton et al. (2012). These data have genotypes at 1000 SNPs, sampled from a total of 215 k variants, for 1107 individual distributed over Eurasia (figure 1). In the comparison we used this data for our five approaches as well.

In the evaluation of the method the distances between true and predicted locations were normalized with the maximum spatial distance between true locations of two *Arabidopsis* assessions (assignment error). For our five methods (NN, GPR-D, GPR-G, GP, DL) we used all 1107 individuals and the same leave-one out approach as for the beech and oak data. Guillot et al. (2016) used a subcollection of 1007 individuals and applied a cross-validation 807 individuals for training and 200 as test individuals.

The assignment errors of our five methods are between those of SPA and SPASIBA, but closer to the best performing SPASIBA (figure 2). For the best 50 % of samples, the NN and GPR-G methods even outperformed SPASIBA.


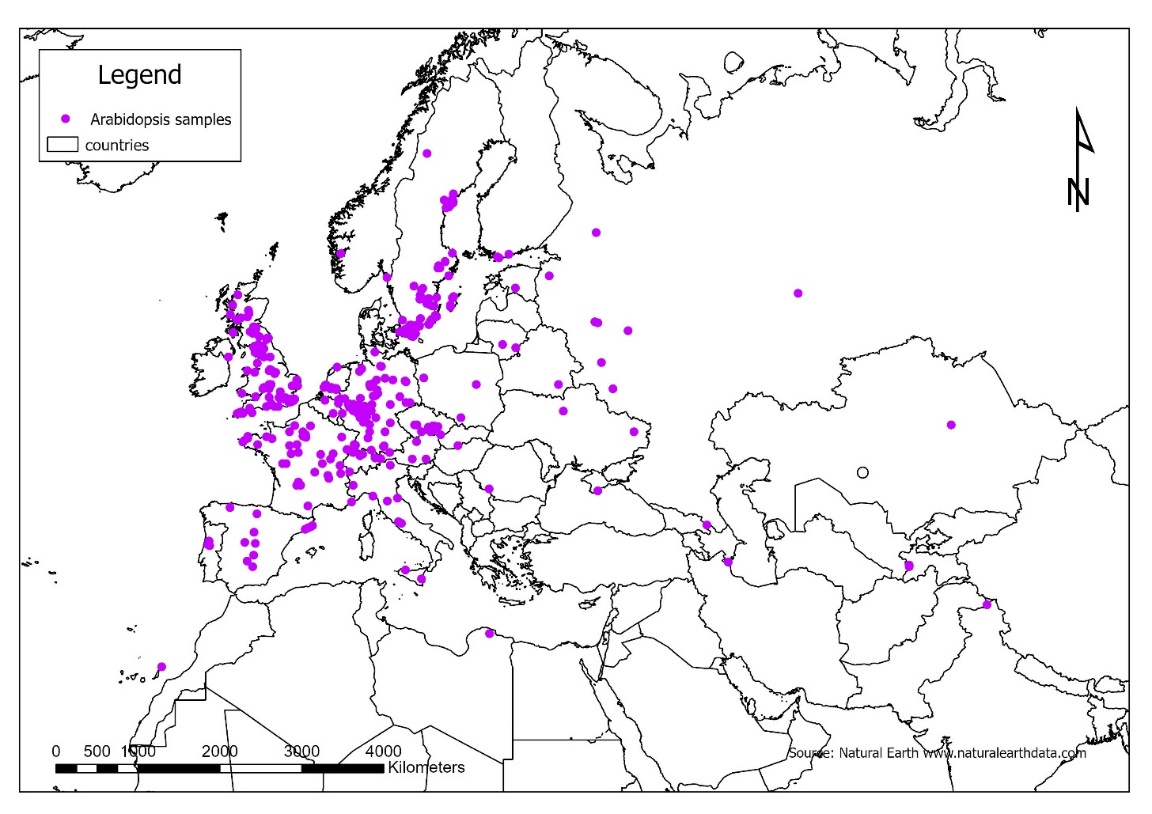


**Figure 1**: Distribution of 1107 individuals of *Arabidopsis thaliana* reported by Horton et al. (2012), shapefile of country borders from [www.naturalearthdata.com](http://www.naturalearthdata.com)


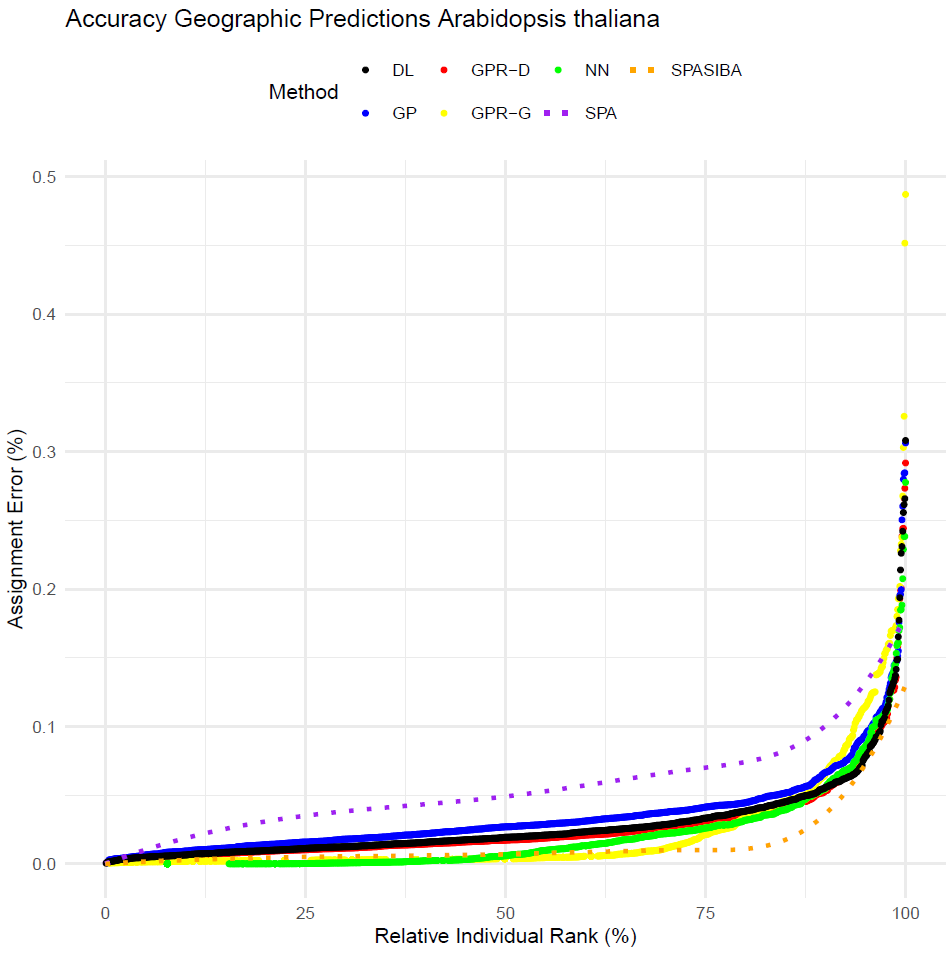


**Figure 2**: Assignment errors estimated using a data set of 1000 sampled SNPs of *A. thaliana*. The errors are expressed as the fraction of the distances between real and predicted locations divided by the maximum distance among individuals. For each approach the individuals are ordered from left to right according to the assignment error.

# References

Guillot G, Jónsson H, Hinge A, Manchih N, Orlando L (2016) Accurate continuous geographic assignment from low- to high-density SNP data. Bioinformatics, 32, 1106-1108.

Horton MW, Hancock AM, Huang YS, Toomajian C, Atwell S, Auton A, Muliyati NW, Platt A, Sperone FG, Vilhjálmsson BJ, Nordborg M, Borevitz JO, Bergelson J (2012) Genome-wide patterns of genetic variation in worldwide *Arabidopsis thaliana* accessions from the RegMap panel. Nature Genet, 44, 212-216.

Wasser SK, Shedlock AM, Comstock K, Ostrander EA, Mutayoba B, Stephens M (2004) Assigning African elephant DNA to geographic region of origin: Applications to the ivory trade. Proceedings of the National Academy of Sciences of the United States of America, 101, 14847-14852.

Yang WY, Novembre J, Eskin E, Halperin E (2012) A model-based approach for analysis of spatial structure in genetic data. Nature Genet, 44, 725-U163.
